# Cerebrospinal fluid glial fibrillary acidic protein provides differential diagnostic value in some forms of dementias

**DOI:** 10.1002/alz.090299

**Published:** 2025-01-09

**Authors:** Pontus Eriksson, Tobias Skillback, Silke Kern, Linus Jönsson, Kaj Blennow, Maria Eriksdotter, Henrik Zetterberg

**Affiliations:** ^1^ Institute of Neuroscience and Physiology, Department of Psychiatry and Neurochemistry, The Sahlgrenska Academy at University of Gothenburg, Sweden, Gothenburg, Västra Götaland Sweden; ^2^ Neuropsychiatric Epidemiology, Institute of Neuroscience and Physiology, Sahlgrenska Academy, Centre for Ageing and Health (AGECAP) at the University of Gothenburg, Gothenburg Sweden; ^3^ Department of Health Sciences Research, Mayo Clinic, Rochester, MN USA; ^4^ Department of Psychiatry Cognition and Old Age Psychiatry, Sahlgrenska University Hospital, Mölndal, Göteborg Sweden; ^5^ Sahlgrenska Academy, University of Gothenburg, Gothenburg Sweden; ^6^ Department of Psychiatry and Neurochemistry, Institute of Neuroscience and Physiology, University of Gothenburg, Mölndal, Vastra Gotaland Sweden; ^7^ Sahlgrenska University Hospital, Mölndal Sweden; ^8^ Karolinska Institutet, Solna, Stockholm Sweden; ^9^ Karolinska Institutet, Dept for Neurobiology, Care Sciences and Society, Center for Alzheimer Research, Div of neurogeriatrics, Solna Sweden; ^10^ H. Lundbeck A/S, Copenhagen Denmark; ^11^ Sahlgrenska University Hospital, Gothenburg Sweden; ^12^ University of Gothenburg, Gothenburg Sweden; ^13^ Department of Neurodegenerative Disease, UCL Queen Square Institute of Neurology, University College London, London UK; ^14^ Institute of Neuroscience and Physiology, Sahlgrenska Academy, Centre for Ageing and Health (AGECAP) at the University of Gothenburg, Gothenburg Sweden; ^15^ Institute of Neuroscience and Physiology, Department of Psychiatry and Neurochemistry, The Sahlgrenska Academy at University of Gothenburg, Mölndal Sweden; ^16^ Karolinska Institutet, Stockholm Sweden; ^17^ University of Science and Technology of China, Hefei, Anhui China; ^18^ Paris Brain Institute, ICM, Pitié‐Salpêtrière Hospital, Sorbonne University, Paris France; ^19^ Stark Neurosciences Research Institute, Indiana University School of Medicine, Indianapolis, IN USA; ^20^ Neurodegenerative Disorder Research Center, Institute on Aging and Brain Disorders, University of Science and Technology of China and First Affiliated Hospital of USTC, Heifei China; ^21^ Universitat Pompeu Fabra, Barcelona Spain; ^22^ Theme Inflammation and Aging, Karolinska University Hospital, Stockholm Sweden; ^23^ Division of Clinical Geriatrics, Department of Neurobiology, Care Sciences and Society, Karolinska Institutet, Stockholm Sweden; ^24^ Wisconsin Alzheimer’s Disease Research Center, University of Wisconsin School of Medicine and Public Health, Madison, WI USA; ^25^ Department of Neurodegenerative Disease and UK Dementia Research Institute, UCL Institute of Neurology, Queen Square, London UK; ^26^ Department of Psychiatry and Neurochemistry, Institute of Neuroscience and Physiology, The Sahlgrenska Academy, University of Gothenburg, Mölndal, Gothenburg Sweden; ^27^ Theme Aging, Karolinska University Hospital, Stockohlm Sweden; ^28^ Hong Kong Center for Neurodegenerative Diseases, Hong Kong China; ^29^ Roche Diagnostics GmbH, Penzberg Germany; ^30^ UK Dementia Research Institute at UCL, London UK

## Abstract

**Background:**

Glial fibrillary acidic protein (GFAP) is a marker of cerebral astrogliosis and occasionally elevated in patients with dementia. GFAP in cerebrospinal fluid (CSF), is routinely requested in referrals to neurochemistry laboratories; however, its ability to differentiate dementias and diagnostic capability is unclear. Our aim was to elucidate this, using two large datasets.

**Method:**

First, GFAP data measured since 2015 was retrieved from the database of the Clinical Neurochemistry Laboratory at the Sahlgrenska University hospital. We then cross‐referenced with the Swedish dementia registry (SveDem). Here, information on ten different diagnoses such as early onset AD (EAD [<65 years]), late onset AD (LAD [≥65 years]), Parkinson disease with dementia (PDD), vascular dementia (VaD) and frontotemporal dementia (FTD), each with specific diagnostic criteria, were retrieved.

The GFAP data was log_10_‐transformed, followed by an analysis of covariance (ANCOVA) and a subsequent post‐hoc Tukey’s test, with GFAP as dependent variable, diagnosis as independent variable and sex and age as covariates.

**Result:**

In total, 1912 individuals (mean [SD] age, 71.9 [8.2] years; 52% male), were included. Lower log_10_‐transformed GFAP concentrations were seen in PDD (mean [SD], 2.68, [0.28] pg/mL), than in EAD, LAD, VaD and FTD; here, mean concentrations of 2.76 (0.24), 2.89 (0.23), 2.89 (0.32) and 2.76 (0.25) pg/mL were observed, respectively.

In the post hoc analysis, GFAP differentiated VaD from EAD (p<0.001). PDD concentrations were significantly different from VaD (p<0.001) and LAD (p<0.001). Further, it also differentiated FTD from VaD (p=0.006) and LAD (p=0.001).

**Conclusion:**

CSF GFAP could on a group level help differentiate VaD from EAD, FTD and PDD. Also, it could differentiate PDD from LAD. These results bear potential clinical relevance, where clinicians in some uncertain cases could use this marker as a differential tool.

